# The Impact of Different Dietary Patterns on Mortality and Prognosis After Non-Metastatic Prostate Cancer Diagnosis: A Systematic Review

**DOI:** 10.3390/healthcare13172201

**Published:** 2025-09-02

**Authors:** Thaw Htet, Florence Cheng, Uhjin Yang, Athulya Harikrishna, Veronica Preda, Juliana Chen

**Affiliations:** 1 Faculty of Medicine, Health and Human Sciences, Macquarie University, Sydney, NSW 2109, Australia; thaw.htet@mq.edu.au (T.H.);; 2 Discipline of Nutrition and Dietetics, Susan Wakil School of Nursing and Midwifery, Faculty of Medicine and Health, The University of Sydney, Sydney, NSW 2006, Australia; 3 Charles Perkins Centre, The University of Sydney, Sydney, NSW 2006, Australia

**Keywords:** dietary patterns, diet, dietitian, prostatic neoplasms, prostate cancer, mortality, prognosis, quality of life

## Abstract

**Objective**: The aim of this systematic review was to compare the impact of various dietary patterns on cancer mortality, recurrence, remission, quality of life, and prostate-specific antigen (PSA) in non-metastatic prostate cancer patients. **Methods**: Ovid Medline, EMBASE, Cochrane Central Register of Controlled Trials (CENTRAL), and Scopus databaseswere searched from inception to March 2024. Dietary interventions or observational studies investigating dietary patterns in men with non-metastatic prostate cancer with at least one primary outcome related to mortality, recurrence, remission, quality of life or PSA/PSA doubling time were included. Two independent reviewers conducted article selection, data extraction, and quality assessment. **Results**: Sixteen eligible articles were included. Adherence to a Mediterranean dietary pattern was linked to lower overall mortality and increased quality of life and adherence to a Prudent diet was associated with both lower overall and cancer-specific mortality risk. A plant-based dietary pattern is associated with increased quality of life. Contrastingly, a Western diet was associated with a higher cancer-specific mortality and overall mortality and high-inflammatory, hyperinsulinaemic, and insulin-resistant diets with increased recurrence. **Conclusions**: Despite the heterogeneity and inconsistencies of PCa literature, there is fair evidence that suggests unprocessed foods with healthier dietary patterns of Mediterranean and prudent diets confer a beneficial effect on overall and cancer-specific mortality, recurrence, and quality of life whereas, a more Western and unhealthier diet generates the opposite. The increased risk of bias prevents conclusive interpretation of these results and, hence, detracts from its clinical implementation. Future research should focus on increasing sample sizes and robustness and standardisation in study design.

## 1. Introduction

Prostate cancer (PCa) is the fourth most commonly diagnosed cancer globally and the eighth leading cause of death, as per the International Agency for Research On Cancer, World Health Organisation [1]. It is the second most common cancer diagnosis (7.1%) and the fifth leading cause of cancer mortality in men [2]. Some countries have demonstrated a decreasing and recently stabilising trend in PCa mortality in the last decade, which is largely owed to the adoption of advanced interventional therapy and recalibration of prostate-specific antigen (PSA) testing recommendations [3,4,5]. However, several Asian and South American countries demonstrate a rising mortality rate, which is thought to be associated with the growing prevalence of obesity and unhealthy diets [3,4]. Overall, the western countries of the Australia and New Zealand region, North America, Western and Northern Europe, along with the Caribbean have the highest incidence of prostate cancer [3].

While genetic factors, ethnicity, family history, hormonal factors, and age are well recognised risk factors for prostate cancer (PCa), evidence suggests that certain dietary patterns may also be associated with PCa [3]. Dietary patterns, defined as habitual food and fluid consumption with an appreciable composition, have been an area of increasing interest in the diet-related cancer literature. These are shown to have a significant role in the physiology associated with cancer development and hence are being more widely explored as a potentially pivotal component in preventative and interventional medicine [6,7]. The association between dietary pattern and PCa is considered to be mediated through the impact on inflammatory and hormonal pathways, including Insulin-Like Growth Factor-1 [8,9], among other possible biochemical pathways [10]. Multiple reviews have also revealed a positive impact on the prognosis with consumption of carotenoids, lycopene, selenium, vitamin E, and soy [11,12,13,14,15].

Currently, PCa literature predominantly explores diet as a preventative means and focuses on individual nutrients or foods [12,13,14,15]. However, dietary intervention is not considered an integral part of the current PCa treatment guidelines [16]. The current mainstay curative treatment of advanced PCa involves surgical removal, radiation, or androgen deprivation therapy (ADT), which commonly have long-term urinary, gastrointestinal, and sexual complications and side effects such as anaemia, bone density loss, and vasomotor flushing [17,18]. ADT is one of the most common PCa interventions and is associated with adverse changes in body composition and increased risk of visceral obesity, diabetes, cardiovascular disease, and insulin resistance [18,19]. The high incidence of PCa and the severity of treatment side effects renders it important that we investigate significant root cause lifestyle factors such as diet [10].

Therefore, this systematic review intends to fill the research gap by conducting a robust comparison across the literature to compare the impact of various dietary patterns on post-diagnostic non-metastatic PCa mortality, recurrence, remission, quality of life, and PSA/PSA doubling time (PSAdt). A secondary outcome was the level of adherence to the different dietary patterns.

## 2. Materials and Methods

### 2.1. Search Strategy

The systematic review followed the Preferred Reporting Items for Systematic Reviews and Meta-Analysis (PRISMA) statement [20] and Cochrane Handbook for Systematic Reviews of Interventions [21]. A preliminary scoping search was conducted on Ovid Medline to identify key terms and phrases prominent in the current literature. A search strategy was then developed with the assistance of an experienced librarian. The protocol of this systematic review was registered with PROSPERO (CRD42022337638).

Formal electronic searches were run in the English language using MeSH terms, keyword, and text words in the Ovid Medline, EMBASE, Cochrane Central Register of Controlled Trials (CENTRAL), and Scopus databases from inception to March 2024. A sample of the complete EMBASE search has been provided in Supplementary Table S1. Relevant additional publications were retrieved via hand searches of reference lists, reviews, PCa guidelines, and clinical trial registries. All references were compiled in EndNote 20 for the removal of duplicates and screening.

### 2.2. Eligibility Criteria

The inclusion criteria included male adults with non-metastatic PCa. This includes patients with localised, non-metastic PCa who had undergone primary therapies such as radical prostatectomy, radiation therapy, or ADT, who did not undergo primary therapy but were under active surveillance and patients with non-metastatic, castration-resistant PCa who had biochemical relapse after primary treatment. Randomised controlled trials (RCTs), non-RCTs, pre-post studies, all other intervention studies, and observational studies (cohort studies, case–control, and cross-sectional studies) were considered. The interventions of interest were dietary patterns studied or prescribed at both an individual or population level. Systematic reviews, other literature reviews, case-series studies and case reports were excluded. All articles were restricted to those relating to humans and those written in the English language.

The primary outcomes studied include mortality (overall and cancer-specific survival reported), recurrence (time to disease progression or progression-free survival), quality of life (score), and PSA (ng/mL)/PSAdt (months). Secondary outcomes included any adherence-to-diet measurements. Controls could be defined as usual therapeutic interventions, no specified dietary pattern, different intensities of dietary exposure, or minimal dietary intervention exposure.

### 2.3. Screening, Selection of Studies, and Data Extraction

**Figure 1:**
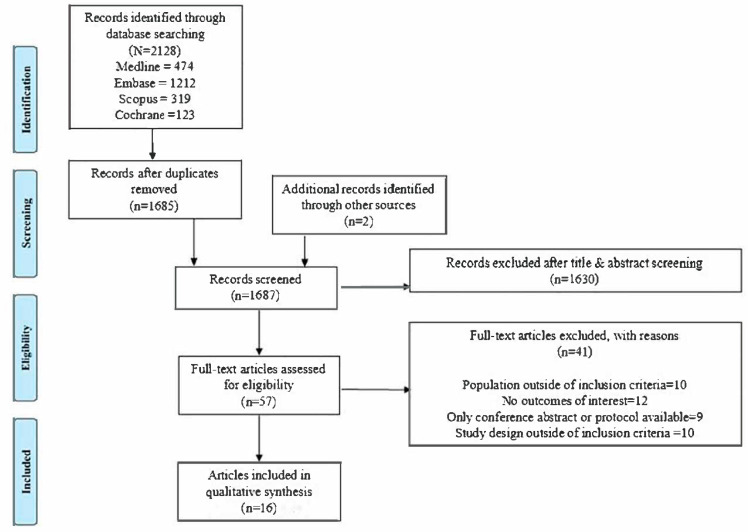
PRISMA Flowchart of Selected Articles.

Screening by title and abstract and full text and selection of studies were performed by two independent reviewers (FC, LY). Any discrepancies were resolved by an arbitrator where required (AH). A PRISMA flowchart of the screening results is presented in Figure 1.

Data extraction was conducted using the Cochrane Handbook data extraction checklist, and all relevant information was tabulated. This information included study details, method design and follow-up duration, participants and cohort, interventions, outcomes, information for risk of bias (RoB) assessment, and conclusions.

### 2.4. Quality Assessment

The Cochrane tool for assessing Risk of Bias (RoB2.0) [22] was used to assess the quality of RCTs and the NIH quality assessment tool for observational studies and pre-post studies [23]. These tools were used to assess the likelihood of bias generated by intervention randomisation, selection of participants, adherence and deviations from intended interventions, data completeness, measurement, and reporting. The selected studies were then stratified into low risk, moderate risk, serious, critical risk of bias, or no information.

### 2.5. Data Synthesis

Data were evaluated using the PRISMA guidelines and were synthesised using a narrative approach.

## 3. Results

### 3.1. Study Selection

The database search yielded 2128 electronic results with 1685 papers remaining after duplicates were removed (Figure 1). An additional two papers were identified through hand searches of reviews, reference lists, and PCa guidelines. After screening the 1687 articles by title and abstract, 57 articles were screened for full text against the inclusion and exclusion criteria (the reasons for exclusion are listed fully in Supplementary Table S2). Sixteen articles were deemed eligible to be included in this review.

### 3.2. Characteristics of Selected Articles

The sixteen articles consisted of seven reporting on RCTs [24,25,26,27,28,29,30], four articles on observational cohort studies [31,32,33,34] and five articles on pre-post trials [35,36,37,38,39], which were published in America (*n* = 13), Australia (*n* = 2), UK (*n* = 1) that studied non-metastatic adult male PCa patients aged 18 years old or older (Table 1). Sample sizes ranged from 7 to 4538 participants. Intervention durations varied from 4 weeks to 24 months. The modes of intervention delivery included regular face-to-face or telephone-based consultations with dietitians, diet guidelines and instructional pamphlets, prepared meals, and prescribed supplementation. Eleven articles implemented dietitian [24,25,26,27,28,29,30,35,36,37] and nutritionist [38] counselling. Some interventions were complemented with physical activity such as walking and high-intensity interval training (HIIT) and guided and self-practised mindfulness-based stress reduction (MBSR). The four observational studies derived dietary patterns from assessments of food frequency via an a priori approach (i.e., Mediterranean Diet Score [MDS]) or index-based (i.e., Plantful-Diet Index [PDI], Empirical Dietary Inflammatory Pattern [EDIP], Empirical Dietary Index for Hyperinsulinaemia [EDIH], and Empirical Dietary Index for Insulin Resistance [EDIR]). The relevant outcomes measured in the eligible studies included overall and cancer-specific mortality reported as hazard ratios, quality of life measured through questionnaires (FACIT-G, SF-36, EPIC-26, EPIC-CP, FACT-P, MAX-PC, and Rotterdam symptom checklist), serum PSA (ng/mL), PSAdt (months), and recurrence reported as hazard ratios. Mean baseline PSA was reported in five articles, baseline PSAdt in four, and baseline PSA range in three articles. None of the eligible articles measured remission. Adherence to dietary pattern was assessed across eight articles via tools such as MEDAS scores, food frequency questionnaires (FFQ), and % of macronutrient consumption retrieved via food records or dietitian follow-ups.

### 3.3. Risk of Bias

**Figure 2:**
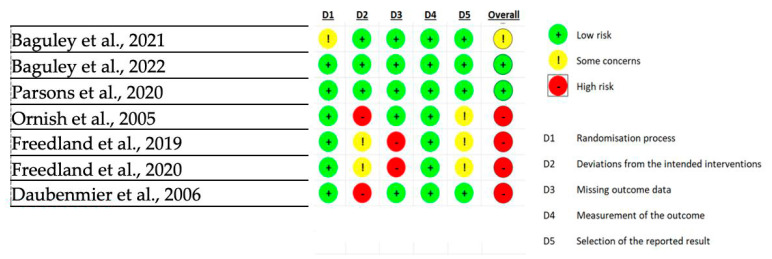
Risk of Bias of RCTs assessed via RoB2.0 tool [24,25,26,27,28,29,30].

Risk of Bias ratings of included articles are presented in Figure 2 and Table 2. From the Cochrane’s RoB2.0 tool, four RCTs were found to have high risk [27,28,29,30], one RCT had some concerns [24], and two RCTs had low risk of bias [25,26]. Two studies were graded as having a high risk of bias due to missing data due to interim analysis showing futility [27,30], and two for deviations of the control group from the intended interventions, with a high percentage of patients making significant changes to their diet despite being allocated null intervention [28,29]. One RCT was reported to have some concerns of RoB due to lack of reporting of randomisation process concealment prior to enrolment [24].

The NIH quality assessment tool was used to assess observational cohort studies and pre-post studies. Of the four cohort studies, one study [31] was rated fair in quality due to study design, which relied on a one-time food frequency questionnaire (FFQ) to grade adherence to a Mediterranean dietary pattern. The other three observational studies were rated good quality [32,33,34]. All five pre-post studies were rated poor in quality [35,36,37,38,39]. This was mainly due to small sample size, inability to blind participants to intervention exposure, substandard reporting of outcomes, and lack of repeat measures taken before, during, and after the intervention. It was also noted that there was a lack of participant compliance to the prescribed diet outlines.

### 3.4. Dietary Patterns

The primary and secondary outcomes from included articles across the different dietary patterns are presented in full in Table 3.

#### 3.4.1. Mediterranean Diet

The review captured three articles (two studies) that demonstrated a positive association between adherence to a Mediterranean diet to improved quality of life and cancer-specific and overall mortality [24,25,32]. A prospective cohort study by Kenfield et al. [32] demonstrated association between a higher Med-Diet adherence score (Med-Diet score (MDS) 6–9) with lower overall mortality (HR: 0.78; 95% CI: 0.67–0.90; *p* = 0.0007) compared to the low adherence group (MDS 0–3). Furthermore, it showed that a 2-point increase in post-diagnostic MDS was linked to a 10% lower risk of overall mortality (HR: 0.90; 95% CI: 0.85–0.96). However, no statistically significant association was found between MDS and cancer-specific mortality.

Baguley et al.’s study [24,25], reporting on 12- and 20-week measures, demonstrated that high adherence to a dietitian-supported Mediterranean diet for 12 weeks generated a statistically significant increase in FACIT-G score as compared to usual care. (I: +9.2; 95% CI: 2.7, 15.8; *p* = 0.006). At 12 weeks, HIIT was added in the intervention arm, which also demonstrated statistically significant improvement in FACIT-G quality-of-life scores at 20 weeks as compared to baseline *p* = 0.032 but was not when compared to 12 weeks or the control arm. Furthermore, no significant differences between groups were found in SF-36 scores at 12 or 20 weeks, though there were improvements when analysing both vitality (+16.2 points; 95% CI: 6.2, 26.2; *p* = 0.002) and mental health composite domains (+4.1 points; 95% CI: 0.1, 8.0; *p* = 0.042) at 20 weeks as compared to usual care.

#### 3.4.2. Plant-Based Diet

Three articles suggested a positive relationship between adherence to a plant-based diet and improved PSA/PSAdt [35,36,37]. Saxe et al.’s 2001 study in non-metastatic, recurrent PCa post-radical prostatectomy [35] demonstrated that a 4-month intervention encouraging plant-based, high-fibre, low-saturated-fat dietary pattern with mindfulness-based-stress-reduction (MBSR) increased mean PSAdt (95% CI) from 8.15 months (3.16–13.1) to 18.2 months (7.2–29.3) in eight patients. Another study conducted for 6 months in non-metastatic, recurrent PCa post-radical prostatectomy or radiation therapy by Saxe et al. [36] that revealed absolute reduction in PSA in four participants and increased PSAdt in nine of ten total evaluable participants from a median of 11.9 (range, 5.4–50.5) months to 112.3 (range, doubling time 8.9-halving time 10.7) months.

Further analysis provided by Nguyen et al. [37] demonstrated that the non-metastatic PCa patients with biochemical recurrence after surgery or radiation therapy achieved a statistically significant decrease median rate of PSA rise from 0.059 (0.014–0.129) pre-study to −0.002 (0.096–0.079) (*p* < 0.01) after six months on plant-based diet. A nonsignificant increase was also measured from 3 to 6 months as compared to the rate at 0–3 months (+0.029). In Loeb et al.’s 2024 observational study the of Health Professionals Follow-Up cohort [33], there were significant improvements in the sexual function (+6.6%) (*p* < 0.0001), urinary incontinence (+10.9%) (*p* < 0.0001), and hormonal/vitality domains (+9.4%) (*p* = 0.04) in the fully adjusted models in patients that scored higher in plant-based diet indices on FFQ. A higher healthful plant-based index score also significantly improved sexual function (+8.4%) (*p* < 0.0001) and bowel function (+12%) (*p* = 0.02) QoL domains.

#### 3.4.3. High Vegetable Diet

A dietary pattern characterised by high intake of vegetables was investigated by Parsons et al. [26], which tested a 4-phase telephone-based dietitian counselling intervention on time to progression(TTP), defined as the time needed for PSA to rise >10 ng/mL, PSAdt <3 years, or increase in tumour grade or size in early stage PCa patients on active surveillance. This randomised trial revealed that there was no significant difference in TTP when comparing intervention vs. control group over 24 months HR 0.97 (95% CI 0.76–1.25) (*p* = 0.84).

#### 3.4.4. Vegan Diet

A vegan soy-supplemented diet was also examined in Ornish D et al.’s study [27], which involved prescription of a 12-month intensive programme, which demonstrated a modest but statistically significant decrease in PSA (ng/mL) in the intervention group −0.25 ± 1.2 *p* = 0.035, whereas the control group, which followed their physicians’ advice for lifestyle changes experienced an increase of 0.38 ± 1.3 (*p* = 0.016). Six from the control group were required to undergo primary treatment due to a rise in PSA or disease progression compared with none in the intervention group. Daubenmier et al. [30] analysed the QoL scores in patients under active surveillance, randomised to a low fat vegan diet, comparing exercise and stress management versus no lifestyle intervention. While QoL scores were high in the intervention group from baseline to post-study, no significant changes at 12 months as compared to the baseline were found in either arm.

#### 3.4.5. Low Fat Diet

Spentzos et al. conducted a two-step pre-post study [38], in non-metastatic PCa patients who had undergone surgery or radiation therapy, which demonstrated a low-fat (<15% of total calorie intake) and high-soy diet, implemented via monthly consultations with a nutritionist and vitamin and soy supplementation added at PSA progression, generated a trend towards longer PSAdt. Median estimated PSAdt was 7.6 (IQR 5.3–13.8) months at step 1, and 11.3 (IQR: 6.7–39.5) at step 2 (*p* = 0.06). The median prolongation in PSAdt was 5 months (95% CI −1.1, 20.9). In total, 10 of 17 patients who progressed to step 2 demonstrated prolonged PSAdt as compared to step 1, which is not of statistical significance.

Thomas et al.’s pilot pre-post study [39] demonstrated that nutritionist advice encouraging a low-saturated-fat diet, rich with fruits and vegetables with mineral and vitamin supplementation increased mean PSAdt from 22.2 to 67.7 months in 7 of the relevant patients. No changes in QoL measured via the Rotterdam Symptom checklist or dietary compliance were reported.

#### 3.4.6. Low Carbohydrate Diet

Freedland et al. conducted a study [28] in 2019, which demonstrated no conferred benefit of a 6-month trial of a dietitian-mediated low-carbohydrate (≤20 carbohydrates/day) dietary pattern and 30 min/day of walking 5 times/week on ADT-initiated patients with regard to PSA levels compared with the control group at baseline, 3 months, and 6 months. The underpowered study was discontinued early due to slow accrual.

Another randomised study by Freedland et al. in 2020 [29] tested a similar dietary and lifestyle intervention post primary treatment. A two-step interim analysis revealed treatment futility and was, hence, discontinued after 45 of the 60 patients had completed the study. A post hoc analysis adjusted for multivariable factors to account for weight-loss-induced haemoconcentration did not show any significant difference in PSAdt between the intervention group and the control group (26 vs. 16 months, *p* = 0.206). However, when adjusted for baseline PSA, pre-study PSAdt, treatment, and haemoconcentration, PSAdt was lowered significantly in the intervention group (30 vs. 13 months, *p* = 0.007).

#### 3.4.7. Prudent vs. Western Diet

One cohort study by Yang et al. [31], which analysed non-metastatic PCa patients enrolled in the Physicians’ Health Study I and II via a food-frequency questionnaire taken at baseline demonstrated a positive association between a Prudent diet and decreased overall mortality [HR: 0.64 (0.44–0.93)] *p* = 0.02 in the highest Prudent quartile. No statistically significant relationship was found between Quartile 4 vs. Quartile 1 of the Prudent diet in cancer-specific mortality.

Conversely, the study also demonstrated an association between high adherence to a Western dietary pattern (Quartile 4) and higher cancer-specific mortality [HR: 2.53 (1.00–6.42)] (*p* = 0.02) and overall mortality [HR: 1.6 (1.16–2.42)] (*p* = 0.01) as compared to those in the lowest quartile (Quartile 1).

#### 3.4.8. High Inflammatory, Hyperinsulinaemic, and Insulin-Resistant Diets

Langlais et al.’s 2022 index-based observational study of the Cancer of the Prostate Strategic Urologic Research Endeavor [34] revealed a positive association between high-inflammatory, hyperinsulinaemic, and insulin-resistant dietary patterns (Empirical Dietary Inflammation Pattern, Empirical Dietary Index for Hyperinsulinemia, and Empirical Dietary Index for Insulin Resistance) and decreased time to prostate cancer progression defined as biochemical recurrence, secondary treatment, or bone metastases. [(HR, 1.27; CI, 1.17–1.37), (HR, 1.24; CI, 1.05–1.46), (HR, 1.22; CI, 1.00–1.48), respectively]. There was no significant association between these diets and prostate cancer-specific mortality.

## 4. Discussion

This systematic review found that the Mediterranean and plant-based dietary patterns are associated with a lower mortality rate, increased quality of life, and decreased recurrence after non-metastatic prostate cancer diagnosis. This in keeping with the recent studies, which have reported that plant-based diet, Mediterranean diet, and a diet rich in cruciferous vegetables have had beneficial effects on Men’s health, including prostate cancer [40,41]. Animal studies showed that upregulation of inflammatory cytokine pathways such as Interleukin-6 (IL-6), IL-8, and Tumour Necrosis Factor (TNF)-α is linked to PCa tumour growth [42,43]. Diets high in vegetables and fruit and low in fat have been shown to improve inflammatory markers such as C-reactive protein and IL-6 [44].

Moreover, there is evidence that active metabolites from fibre, flavonoid, carotenoids, and phenolic acid in a plant-based diet have anticancer effects and reduce cancer mortality via antioxidant and anti-inflammatory actions [45,46]. There are also studies that showed that Indole-3-carbinol in cruciferous vegetables can inhibit the growth of human prostate cancer cells [47]. Hence, the conferred benefit of Mediterranean, Prudent, and plant-based diets on overall mortality in cancer is thought to be linked to anti-inflammatory effects of higher fruit, vegetable, and reduced saturated fat consumption [10]. However, while studies of the Mediterranean diet were high in quality with a low risk of bias, the plant-based diet studies varied in quality with most studies showing high risk of bias.

Previous literature also showed that a diet consisting of different types of soy food reduces the risk of PCa by 30% and reduces PSA levels [48,49]. While the exact mechanism of soy-based diet on the pathophysiology of PCa is not clear, there is some evidence that showed that soy intake was inversely related to serum testosterone level in the Japanese population, and an isolated soy protein-based diet can reduce the development of progress of prostate neoplasm in animals [50,51,52]. Two studies, which were included in our review, where patients were on a vegan soy-supplemented diet and low fat, high soy-supplemented diet, however, did not show any reduction in PSA. Both studies were, however, considered to have high risk of bias due to deviation from the intended interventions.

Across the cancer nutrition literature, the validity of biomarkers in cancer prognosis is commonly questioned, and although higher quality dietary patterns may show beneficial trends, results do not demonstrate statistical significance [53,54]. This is reflected in the current review where low-carbohydrate diets and low-fat dietary patterns demonstrate non-statistically significant potential in prolonging PSAdt. It has been shown that cancers including PCa are promoted by increased oxidative stress, and patients with PCa expressed high levels of oxidative stress markers such as gluthathione peroxidase and superoxide dismutase [55]. While a dietary pattern high in vegetables and fruits contains copious amount of antioxidant, wider PCa literature showed no significant or consistent effect on PSA similar to the studies included in our review [56]. Moreover, PSA is reported to be affected by factors such as BMI, ethnicity, age, and urinary tract infections [56]. Hence, without removing these confounding factors, the effect of diet on PSA/PSAdt is likely to be of limited clinical use.

Treatment of PCa, including ADT, can have an impact on both physical and mental health, including an individual’s perception of QoL [57]. Our study showed that there was a general overall trend towards improved QoL scores in patients consuming a post-diagnostic Mediterranean, high-vegetable, or plant-based diet. However, this effect was not consistent or sustained across all studies. Due to the vast definition of QoL, there remains significant heterogeneity in assessment and reporting, which affects the effective evaluation of the degree and nature of conferred benefit.

The two studies investigating Western dietary patterns or diets classified as being high inflammatory, hyperinsulinaemic, and insulin-resistant showed increased mortality rate, decreased quality of life, and increased cancer recurrence. One of these studies, however, only used one food frequency questionnaire for assessment, which limits the scope of the assessment of the clinical outcomes. There are pre-existing studies that link Western diet to increased Body Mass Index (BMI) including obesity, chronic disease, and cancers [58]. This may be due to several factors including chronic excessive energy intake, increased dietary fat, and meat consumption associated with Western diets [59]. There are also several studies that showed that men with high BMI are at an increased risk of high-grade prostate cancer, poor prognosis, and recurrence [60,61,62]. Increased insulin levels associated with high BMI leads to a reduction in Insulin-Like Growth Factor Binding protein and increased the biological activity of IGF-1, which can increase angiogenesis needed for development of PCa via IGF-1 receptor as well as stimulate growth factors such as vascular endothelial growth factor (VEGF) [59,63].

Recent studies suggested that increased fat consumption, in particular saturated fatty acid (SFA), is associated with worse prognosis and aggressive PCa. Diets high in SFA cause disruption of the growth factor signalling pathway and prostate hormonal pathway, in addition to inflammatory pathways, which can then lead to progression of PCa [64,65]. There were also animal studies, which reported that a high fat dietary pattern was associated with progression of PCa by activating macrophage inhibitory cytokine-1 signalling and enhancing IL-6 and IL-8 and histamine signalling pathways [43,66]. Some studies have shown that Western diets high in fat can cause gut dysbiosis, leading to high levels of lipopolysaccharide levels linked with PCa growth [66,67].

Multidisciplinary care is strongly recommended for weight and chronic disease management [68,69]. A key element of coordinated cancer care is the involvement of a multidisciplinary team (MDT). Incorporating a multidisciplinary approach of care may include a combination of dietary counselling, physical exercise, endocrine therapy, mental wellbeing activities, and social support networks. These may augment prognostic improvements in patients with breast cancer post-adjuvant chemotherapy or following long-term tamoxifen use, and ADT-treated PCa patients, such as recurrence risk, overall mortality, and weight and metabolic outcomes, including reduced risk of developing type 2 diabetes [19,70,71]. Moreover, the implementation of dietitian counselling has been shown to improve dietary adherence by improving patients’ diet literacy and increasing motivation and health outcomes [72]. As such, the included studies that incorporated dietitian counselling have demonstrated high adherence, strengthening the associations between dietary patterns and outcomes reported.

Most of the accrued RCTs and pre-post studies had a high risk of bias due to small sample sizes, loss to follow-up, or weak statistical analyses. Of the sixteen studies included, only seven were RCT, while others were observational or pre-post studies. The duration of the studies varied significantly, with the shortest study being only 4 weeks, which limits the clinical implication of the outcomes. The studies included in this review were also of varying sample sizes ranging from 7 to 45,382, which restricts the comparability and generalizability. Some of the studies included varying degrees of physical activities from walking daily to HIIT as well as MBSR, which may have impacted the findings. In addition, interventional methods vary from face-to-face vs. telephone consultations with or without additional provision of pamphlets, meal plans, and supplementation, which makes the interpretation of the results more challenging.

This highlights the practical challenges in nutritional research in cancer due to the scientific standards required to validate results and confer practical implications, such as establishing baseline participant nutrition, isolating nutritional components or composition, blinding of patients to their food intake, gathering sufficient funding, and patient adherence to demonstrate statistically significant change in diseases with long latency progression, rendering it generally incompatible with such standards [73]. The strict exclusion criteria also limited the number of accrued eligible studies, with unfortunate exclusions of some highly robust articles due to the lack of reporting of metastatic status. Restricting the search to papers available in the English language may have also excluded otherwise relevant studies. Furthermore, while a qualitative approach allowed for a broad range of outcomes to be studied, the variable quality and design of the studies included, as well as the clinical heterogeneity of the patients included, restricted any further meta-analyses to be carried out.

Another challenge in nutritional research in cancer patients includes the effect of diet on an individual’s gut microbiomes and vice versa. It has been established that gut microbiomes can differ significantly between individuals, as both genetic factors and the diet of an individual can influence the diversity and abundance of gut microbiomes [74]. There is also evolving evidence in recent years that certain gut microbiomes may influence the effect of cancer outcomes via inflammatory and growth signalling pathways, and manipulating the gut microbiome via certain diets and supplementation may have a role in future cancer treatment strategies [67,75]. The studies included in this review did not include the gut microbiome data, which may impact the clinical outcomes.

The exclusion of metastatic prostate cancer patients allowed us to emphasise long-term health outcomes and patterns of progression, since outcomes of metastatic PCa are starkly different from that of early-stage PCa. However, non-metastatic PCa patients are a clinically heterogeneous group, which includes patients who had primary therapy, who were on active surveillance and had biochemical recurrence after primary therapies with different and variable clinical outcomes and prognoses. It has been shown that the incidence of disease progression was higher in patients in the active surveillance group compared with patients treated with primary therapy, with some studies reporting that 30% of non-met PCa patients under active surveillance can require treatment or may progress within two years [76,77]. Similarly, clinical outcomes and prognoses differ between non-metastatic PCa patients treated with primary therapy and patients with biochemical recurrence, with one recent study reporting median overall survival rates at ten years of 84.8% and 69.7%, respectively [78]. Therefore, interpretation of the results and clinical implications cannot be generalised unless the studies are performed in a similar subgroup of patients.

Overall, our findings showed that higher quality diets including Mediterranean, Prudent, and plant-based diets are associated with improved outcomes similar to findings in wider nutrition literature in other cancers such as breast, colorectal, and ovarian cancers [79], as well as other chronic diseases such as cardiovascular disease and diabetes [80,81]. Conversely, the increased risk of mortality associated with the Western/unhealthy diet may be strongly linked to high red meat, fat, carbohydrate, and sugar intake, which is thought to increase glycaemic load and promote tumorigenesis [10,58,82,83]. Adherence to vegan and low-fat diets indicated a trend towards decreasing PSA and increasing PSAdt; however, no current relevant studies reached statistical significance. However, due to high risk of bias and other limiting factors, driven largely by the common issues in validity faced in nutrition oncology research, these findings should be interpreted with considerable caution. that must be addressed moving forward.

Currently, healthy diet and lifestyle changes are often promoted as a means to augment prognosis, although they are not considered as potential means for PCa-related intervention [16,79,84]. The growing research of dietary patterns continues to give us an insight into the potential for dietary intervention to become part of the management guidelines for PCa [79,84]. Development and adoption of various a priori and index-based approaches in deriving dietary patterns are becoming increasingly better understood and analysed, which is important for strengthening the current body of nutritional research which is heterogeneous.

While this review was conducted with well-defined inclusion and exclusion criteria, which allowed for a robust analysis of the relationship between dietary patterns and prostate cancer prognostic outcomes, including QoL, it also highlights the challenges and limitations in nutritional research, particularly in clinically heterogenous patient groups such as non-metastatic PCa patients with variable prognoses. Hence, it is important to ensure future nutritional studies are designed and conducted to overcome these challenges. Randomised, prospective studies in specific subgroup of patients that are adequately powered would be ideal, although the recruitment and blinding process would still be challenging in cancer nutrition research. In addition to the influence of patients’ basic demographic factors, other patient-related factors such as genetic factors, gut microbiome data, degree of motivation, adherence, and physical activities, as well as disease-related factors including variable clinical outcomes of different patient subgroups—active surveillance vs. intervention vs. biochemical recurrence and the effect of treatments used—need to be considered as confounding factors.

## 5. Conclusions

Our review showed fair evidence that suggests unprocessed foods with healthier dietary patterns may confer a beneficial effect on overall and cancer-specific mortality, recurrence, and quality of life, whereas a more Western and unhealthier diet seems to generate the opposite. Certain diets reported to influence the risk of PCa also appear to influence cancer prognosis. While these beneficial diets may be considered as part of the MDT management guidelines for PCa in the future, further high-quality studies are required to ascertain the true extent of the benefit due to the heterogeneity and inconsistencies of the current literature. This systematic review also reaffirms the demanding nature of nutrition research and further highlights the current scientific landscape, which favours pharmacological studies and causal relationships. However, there is an increasing appreciation that nutrition and lifestyle are pivotal in holistic health, which itself is complex and intricately interconnected. Though there is progress towards standardisation of nutrition studies, there should also be consideration in applying the general processes of scientific validation more flexibly in evaluating this area of research.

## Figures and Tables

**Table 1:** Characteristics of included articles (*n* = 16).

Author, Year, Country, Study Design	Study Population	N Trial Arms/N Total Population	Characteristics	Dietary Pattern Intervention/Observation Design	Duration	A = Retention at the End of Intervention	Outcome Measured
Baguley et al., 2021, Australia, RCT [24]	Non-metastatic PCa patients ADT for ≥3 months BMI 18.5–34.9 kg/m^2^ Age ≥ 18 years Non-smoker/quit smoking for the previous ≥3 months.	I: 12 C: 11	Age: 65.9 ± 7.8 yr PSA: 1.1 ± 1.3 ng/mL Treatment: ADT duration: 33.8 ± 35.6 months)	Mediterranean Diet I: 30–45 min face-to-face consultation with dietitian every 2 weeks for 12 weeks. C: Usual care for 12 weeks	Active intervention: 12 weeks. Measurements at baseline, 8, and 12 weeks.	Intervention A = 92% (11 out of 12) (1 dropped out due to metastasis) Control A = 91% (10 out of 11) (1 dropped out due to personal reasons)	Quality of life, adherence
Baguley et al., 2022, Australia, RCT [25]	Non-metastatic PCa patients ADT for ≥3 months BMI 18.5–34.9 kg/m^2^ Age ≥ 18 years Non-smoker/quit smoking for the previous ≥3 months.	I: 12 C: 11	Age: 65.9 ± 7.8 yr PSA: 1.1 ± 1.3 ng/mL Treatment: ADT duration: 33.8 ± 35.6 months)	Mediterranean Diet I: 30–45 min face-to-face consultation with dietitian every 2 weeks for 20 weeks with high intensity interval training (HIIT) starting at 12 weeks (4 × 4 min 85–95% heart rate peak, 3× week). C: Usual care for 12 weeks	Active intervention: 20 weeks. Measurements at baseline, 8, 12, and 20 weeks.	Intervention A = 75% (9 out of 12) (1 dropped out due to metastasis, 2 ineligible to receive HIIT) Control A = 91% (10 out of 11) (1 dropped out due to personal reasons)	Quality of life, adherence
Parsons et al., 2020, America, RCT [26]	Early stage (T2a or less) PCa patients Age 50–80 yr PSA <10 ng/mL On active surveillance	I: 226 C: 217	Intervention Age: 63.7 ± 6.5 yr Median PSA range: >2.5–5 Control Age: 63.5 ± 6.6 yr Median PSA range: >2.5–5	High-Vegetable Diet Intervention: 4-phase telephone-based diet counselling intervention promoting consumption of ≥7 daily vegetable servings Control: Receive published diet and prostate cancer guidelines	24-month follow-up.	Intervention A = 81.7% (183 out of 226) Control A = 79.5% (171 out of 217)	PSA, PSAdt, upgrade in tumour size or grade
Ornish et al., 2005, America, RCT [27]	Stage T1–T2 PCa patients on active surveillance who elected not to have any other conventional PCa treatment No other life-threatening co-morbidities	I: 44 C: 49	Intervention Age: 65 ± 7 yr PSA: 6.32 ± 1.72 Control Age: 67 ± 8 yr PSA: 6.28 ± 1.66	Vegan, soy-supplemented Diet I: Vegan diet supplemented with soy (1 daily serving of tofu plus 58 gm of a fortified soy protein powdered beverage), fish oil (3 gm daily), vitamin E (400 IU daily), selenium (200 mcg daily) and vitamin C (2 g daily), moderate aerobic exercise (walking 30 min 6 days weekly), stress management techniques (gentle yoga based stretching, breathing, meditation, imagery and progressive relaxation for a total of 60 min daily) and participation in a 1 h support group once weekly to enhance adherence the intervention. Registered dietitian was made available for counselling and weekly telephone follow-up by nurse manager for first 3 months C: Follow their physicians’ advice for lifestyle changes.	12 months	Intervention A = 93% (41 out of 44) Control A (C) = 88% (43 out of 49)	PSA, adherence
Freedland et al., 2019, America, RCT [28]	PCa patients who have initiated ADT BMI ≥ 24 kg/m^2^ Symptomatic metastatic excluded	I: 20 C: 22	Intervention Age: 66 (61, 76) yr PSA: 20.8 (7.6, 45.6) Control Age: 66 (56, 70) yr PSA: 18.8 (5.5, 46.5)	Low-Carbohydrate Diet I: 6-month trial of LCD instructed to eat ≤20 g/carbs/day. Dietitian coaching weekly for 0–3 months and then biweekly from 4 to 6 months via telephone or face-to-face consultation to limit carbohydrate intake and provide a list of foods to avoid. Walking ≥30 min/day for ≥5 days/week C: Avoid dietary changes	6 months	A (I) = 55% (11 out of 20) A (C) = 82% (18 out of 22)	PSA
Freedland et al., 2020, America, RCT [29]	PCa patients who have had primary treatment BMI ≥ 24 kg/m^2^ Symptomatic metastatic excluded PSADT 3–36 months	I: 30 C: 27	Intervention Age: 71 (69, 74) yr PSA: 1.5 (0.8, 3.3) ng/mL PSAdt: 11 (7, 16) months Control Age: 72 (65, 74) yr PSA: 2.3 (0.9, 4.7) ng/mL PSAdt: 14 (10,20) months	Low-Carbohydrate Diet I: 6-month trial of LCD instructed to eat ≤20 g/carbs/day. Dietitian coaching via telephone or face-to-face consultation to limit carbohydrate intake and provide a list of foods to avoid. C: Avoid dietary changes	Planned for 6 months but was stopped early for 12 participants due to interim analysis showing futility	A (I) = 87% (26 out of 30) A (C) = 73% (19 out of 27) (Study was discontinued at interim analysis after 45 participants completed the study	PSAdt
Daubenmier et al., 2006, America, RCT [30]	Stage T1–T2 PCa patients on active surveillance who elected not to have any other conventional PCa treatment No other life-threatening co-morbidities	I: 44 C: 49	Intervention Age: 65 ± 7 yr PSA: 6.32 ± 1.72 Control Age: 67 ± 8 yr PSA: 6.28 ± 1.66	Vegan, soy-supplemented Diet I: Vegan diet supplemented with soy (1 daily serving of tofu plus 58 gm of a fortified soy protein powdered beverage), fish oil (3 gm daily), vitamin E (400 IU daily), selenium (200 mcg daily) and vitamin C (2 g daily), moderate aerobic exercise (walking 30 min 6 days weekly), stress management techniques (gentle yoga based stretching, breathing, meditation, imagery and progressive relaxation for a total of 60 min daily) and participation in a 1 h support group once weekly to enhance adherence the intervention. Registered dietitian was made available for counselling and weekly telephone follow-up by nurse manager for first 3 months C: Follow their physicians’ advice for lifestyle changes.	12 months	Intervention A = 93% (41 out of 44) Control A (C) = 88% (43 out of 49)	QoL
Yang et al., 2015, America, Cohort study [31]	Non-metastatic PCa patients enrolled in the Physicians’ Health Study I or II	926	Intervention Age: 68.6 ± 6.9 yr Median PSA range at diagnosis: 4–9.9 ng/mL	Western vs. Prudent One food-frequency questionnaire (FFQ) was sent to all participants between 1999 and 2002. Dietary patterns were derived, and two patterns were identified—Western and Prudent. Each participant was given a “Western” and “Prudent” score.	From FFQ completion until death or end of follow-up	A = 100%; 333 recorded deaths out of 926	Mortality(Cancer-specific and overall)
Kenfield et al., 2014, America, Prospective Cohort study [32]	Non-metastatic PCa patients Stage T3a or lower US male health professionals Age 40–75 yr No other cancers (except melanoma skin cancer)	4538	Baseline in 1986 LA Age in 1990: 52.6 yr MA Age in 1990: 54.3 yr HA Age in 1990: 55.3 yr	Mediterranean Diet Nol active intervention. Semi-quantitative food-frequency questionnaire (FFQ) every 2 years and diet information every 4 years. Med-Diet score was calculated to stratify participants into low adherence (LA) (0–3), moderate adherence (MA) (4–5) and high adherence (HA) (6–9) to Med-Diet.	From diagnosis to lethal outcome or to January 2010	A = 100%	Mortality (Cancer-specific and overall)
Loeb et al., 2024, America, Cohort study [33]	Non-metastatic PCa patients in the Health Professionals Follow-Up Study (1986–2016)	3505	Median age at first FFQ: 75.4 (61.2, 96.7) Most patients had PSA < 10 mg/mL at diagnosis	Plant-Based Diet A semiquantitative FFQ at baseline and then every 4 years thereafter. Data were used to calculate the overall plant-based diet index (PDI) and healthful PDI (hPDI). Participants were then stratified according into 5 quintiles. PDI and hPDI were then cumulatively updated after each participant FFQ after diagnosis.	From FFQ completion until death or end of follow-up	A = 100%	QoL
Langlais et al., 2022, America, Cohort study [34]	Non-metastatic PCa patients in the CaPSURE longitudinal observational cohort with biopsy-proven cancer	2056	Mean age: 64.4 ± 7.9 yr Mean PSA:	High-inflammatory, Hyperinsulinaemic and Insulin Resistant Diets A comprehensive lifestyle questionnaire and full-length FFQ administered at three time points between 2004–2016	From FFQ completion until death or end of follow-up	A = 100%	Time to prostate cancer progression, Cancer-specific mortality
Saxe et al., 2001, America, pre-post study [35]	Recurrent PCa patients confirmed via rising PSA on 2 sequential tests less than 6 months apart after radical prostatectomy as primary therapy Biopsy-confirmed, operable, invasive, non-metastatic PCa	8 (10 studied, 2 were excluded in this review due to metastasis)	Age (Mean of 8 eligible patients): 68 yr PSA: 0.895 ng/mL PSAdt: 8.1375 months	Low-Saturated-Fat, Plant-Based, High-Fibre Diet Intervention: 4-month group-based diet and MBSR intervention; Plant-based diet, low in saturated fat and high in fibre. Individual counselling. 12 × 3–4 h weekly classes with elements of MBSR including mindfulness meditation training, yoga and social support	4 months	A = 100% (8 out of 8)	PSAdt
Saxe et al., 2006, America, pre-post study [36]	Recurrent PCa patients confirmed via rising PSA on ≥3 serial tests at least 1 month apart after radical prostatectomy or radiation as primary therapy Biopsy-confirmed, operable, invasive, non-metastatic PCa	14	Age (mean): 68.2 yr PSAdt mean: 12.4	Plant-based Diet Intervention: 6-month plant-based diet and stress reduction at Moores UCSD Cancer Center via dietary counselling and instructional materials provision weekly then monthly with 10 × 3 h group meetings, cooking class and shared model meal with telephone follow-up counselling. Stress reduction implemented via clinical psychologist and oncology nurse-led group discussions and encouraged to practise taichi, yoga or meditation at least 15 min a day.	6 months	A = 71% (10 out of 14) (1 dropped out before baseline measurements and 1 before 6-month measurement due to difficulty with diet implementation, 2 opted for hormonal therapy)	PSAdt
Nguyen et al., 2006, America, pre-post study [37]	Recurrent PCa patients confirmed via rising PSA on ≥3 serial tests at least 1 month apart after radical prostatectomy or radiation as primary therapy Biopsy-confirmed, operable, invasive, non-metastatic PCa	14	Age (mean): 68.2 yr PSAdt mean: 12.4	Plant-based Diet Intervention: 6-month plant-based diet and stress reduction at Moores UCSD Cancer Center via dietary counselling and instructional materials provision weekly then monthly with 10 × 3 h group meetings, cooking class and shared model meal with telephone follow-up counselling. Stress reduction implemented via clinical psychologist and oncology nurse-led group discussions and encouraged to practise taichi, yoga or meditation at least 15 min a day.	6 months	A = 71% (10 out of 14) (1 dropped out before baseline measurements and 1 before 6-month measurement due to difficulty with diet implementation, 2 opted for hormonal therapy)	Rate of PSA rise
Spentzos et al., 2003, America, pre-post study [38]	Non-metastatic asymptomatic PCa patients who had undergone primary therapy (Radiation or surgery). Enrolled between 1998 and 2001 No other cancers (except melanoma skin cancer)	18	Age [median (range)]: 71 (59–81) yr PSA [median (IQR)]: 11.0 (6.1, 15.0)	Low-Fat, High Soy Diet I: Low-fat diet with goal to reduce fat intake to 15% of daily calories implemented via monthly consultation with a nutritionist. Additionally given 2-month supplies of vitamin E (400 IU/day), selenium tablets (200 mg/day), and multivitamins (Spectravite) Step 2. On PSA progression, soy supplement was added.	Median follow up: 10.5 months	A = 94% (17 out of 18) (17 progressed to step 2, 4 withdrew, 2 demonstrated clinical progression, 3 patient continues with the study)	PSAdt, adherence
Thomas et al., 2003, pre-post study [39]	Progressive PCa patients confirmed via increases in ≥2 tumour markers recruited between March 2001 and Nov 2001	7 out of a total of 37 patients relevant to this review	PSA: 24 ng/mL PSAdt (12 months pre-trial to baseline): 22.2 months	Low Fat Dietary advice to encourage a low saturated fat diet rich in fruits and vegetables, sodium salicylate (350 mg), manganese gluconate (20 mg), copper gluconate (20 mg), vitamin C (400 mg), multiple vitamin and mineral supplement	Mean: 17.2 months follow-up	A = 86% (6 out of 7)	PSAdt, adherence

**Table 2:** Risk of Bias of Observational and Pre-post Studies using the NIH quality assessment tool for observational studies and pre-post studies.

Citation	RoB Rating	Reason
Yang et al., 2015 [31]	Fair	Only one Food Frequency Questionnaire assessed
Kenfield et al., 2014 [32]	Good	-
Loeb et al., 2024 [33]	Good	-
Langlais et al., 2022 [34]	Good	-
Saxe et al. 2001 [35]	Poor	Small sample size. No randomisation or blinding
Saxe et al. 2006 [36]	Poor	Small sample size, loss to follow-up, lack of blinding
Nguyen et al., 2006 [37]	Poor	Small sample size. No randomisation or blinding
Spentzos et al., 2003 [38]	Poor	Small sample size. No randomisation or blinding
Thomas et al., 2003 [39]	Poor	Small sample size, poor statistical analysis, poor reporting of patient population information, unclear eligibility criteria

**Table 3:** Primary and Secondary Outcomes from Included Articles (*n* = 16).

Mediterranean Diet			
Study	Results		Summary of Direction of Change
Kenfield et al., [32] Prospective Cohort study	**Overall mortality** **HA ^#^:** HR: 0.78; 95% CI (0.67–0.90), *p* = 0.0007 **MA:** HR: 0.92; 95% CI (0.80–1.05), *p* = 0.0007 **LA:** 1.00		Highest scoring cohort on Med-Diet score showed 22% lower risk of overall mortality. Also, a 2-point increase in post-diagnostic adherence to Med-Diet score was associated with 10% lower risk of overall mortality (HR: 0.90; 95% CI: 0.85–0.96). Men who consumed ≥5 servings of olive oil/week post-diagnosis had a 31% lower risk of overall mortality (HRL0.69; 95% CI: 0.55–0.88)
	**Cancer-specific mortality** **HA:** HR: 1.01; 95% CI (0.75–1.38), *p* = 0.95 **MA:** HR: 1.06; 95% CI (0.79–1.43), *p* = 0.95 **LA:** 1.00		Higher adherence to Med-Diet demonstrated no association with cancer-specific mortality.
Baguley et al., [24], RCT	**QoL FACIT-G** **Baseline** **I:** 83.1 (78.7, 87.4)**, C:** 82.6 (78.1, 87.2) **12 weeks** **I:** 90.5 (85.9, 95.0), **C:** 81.2 (76.5, 86.0)		Significantly higher FACIT-G score at 12 weeks as compared to baseline +9.2 (2.7–15.8), *p* = 0.038 and as compared to control. (*p* = 0.006)
	**QoL (Baseline-12 wk) SF-36** **Baseline** **I:** 57.2 (47.2, 67.3), **C:** 59.7 (49.2, 70.2) **12 weeks** **I:** 66.3 (55.9, 76.7), **C:** 62.2 (51.3, 73.1), *p* > 0.999		No significant changes at 12 weeks in SF-36 scores in intervention arm as compared to baseline (*p* = 0.710) and control groups at 12 weeks as compared to baseline +4.1 points (−10.9, 19.1) *p* = 0.588
	**Adherence (MEDAS score)** **8 weeks:** **I:** 64% (*n* = 7/11) reaching ≥75% **C:** 0% (*n* = 0/10) reaching ≥75% **12 weeks:** **I:** 81% (*n* = 6/9) reaching ≥75% **C:** 10% (*n* = 1/10) reaching ≥75%		High adherence to Med-Diet as compared to usual care group at 8 weeks [+3.5, (2.4, 4.5); *p* < 0.001] and 12 weeks [+4.9, (3.8, 5.9); *p* < 0.001], and increased adherence to Med-Diet in intervention group from 8 to 12 weeks
Baguley et al., [25], RCT	**QoL (Baseline-20 wk):** FACIT-G **Baseline** **I:** 83.1 (78.7, 87.4), **C:** 82.6 (78.1, 87.2) **12 weeks** **I:** 90.5 (85.9, 95.0), **C:** 81.2 (76.5, 86.0) **20 weeks** **I:** 91.3 (86.4, 96.3), **C:** 86.5 (81.8, 91.3)		Statistically significant improvement in intervention arm with diet and HIIT as compared to baseline *p* = 0.032, but not compared to 12 weeks *p* > 0.999 or control arm *p* = 0.167.
	**QoL (Baseline-20 wk):***SF-36* **Baseline** **I:** 57.2 (47.2, 67.3), **C:** 59.7 (49.2, 70.2) **12 weeks** **I:** 66.3 (55.9, 76.7), **C:** 62.2 (51.3, 73.1) **20 weeks** **I:** 59.7 (48.5, 70.9), **C:** 62.9 (52.0, 73.8)		No significant differences between groups at 12 [+4.1 (−10.9, 19.1); *p* = 0.588] or 20 weeks [−3.1 (−18.7, 12.4); *p* = 0.688].
	**Adherence (MEDAS) I as compared to C 12 weeks:** **I:** 81% (*n* = 6/9) reaching ≥75% **C:** 0% (*n* = 0/10) reaching ≥75% **20 weeks** **I:** 66% (*n* = 6/9) reaching ≥75% **C:** 0% (*n* = 0/10) reaching ≥75%		Increased adherence to Med-Diet overall in the intervention group as compared to usual care at 12 weeks I: +4.9, (3.8, 5.9); *p* < 0.001 and 20 weeks [+2.8 (1.27, 4.45); *p* = 0.001.
**Plant-Based**			
**Study**		**Results**	**Summary of Direction of Change**
Saxe et al., [35], pre-post study		**PSAdt ^ Mean (95% CI)** **Pre-study:** 8.15 (3.16–13.1) **4 months:** 18.2 (7.2–29.3)	PSAdt mean in months (95% CI) increased from 8.15 (3.16–13.1) to 18.2 (7.2–29.3). 2 non-metastatic patients had a decrease in absolute PSA. The rate of PSA increase decreased in 7 of 8 non-metastatic patients.
Saxe et al., [36], pre-post study		**PSAdt Median** **Pre-study to baseline:** 11.9 (5.4–50.5) **Baseline-6 months:** 112.3 (−10.7–8.9)	Nine of 10 patients showed elongation of PSA doubling times compared. Four of these 9 patients experienced an absolute reduction in their PSA levels over the entire 6-month study. Median PSA doubling time increased from 11.9 months (prestudy) to 112.3 months (intervention).
Nguyen et al. [37], pre-post study		**Rate of PSA rise median (range)** **Pre-study:** 0.059 (0.014 to 0.129) **0–3 months:** –0.002 (–0.096 to 0.079) **0–6 months:** 0.029 (–0.067 to 0.136)	Significant decrease in PSA rate rise (*p* < 0.01). The negative value indicates a median reduction in absolute PSA.
Loeb et al., [33], cohort study		**QoL EPIC-PC** **PDI (%Diff Quintile 5 vs. Quintile 1)** **Sexual function:** 6.6 (*p* < 0.0001) **Urinary irritation:** 10.9 (*p* < 0.0001) **Urinary incontinence:** 11.8 (*p* = 0.01) **Bowel function:** 8.3 (*p* = 0.34) **Hormonal/vitality:** 9.4 (0.04) **hPDI (%Diff Quintile 5 vs. Quintile 1)** **Sexual function:** 8.4 (<0.0001) **Urinary irritation:** 0.8 (*p* = 0.85) **Urinary incontinence:** 7.7 (*p* = 0.15) **Bowel function:** 12 (*p* = 0.02) **Hormonal/vitality:** 4.6 (*p* = 0.35)	There was significant QoL improvements PDI Quintile 5 as compared to Quintile 1 in the sexual function, urinary irritation, urinary incontinence and hormonal/vitality domains in the fully adjusted model and there were significant improvements in hPDI Quintile 5 vs. Quintile 1 in the sexual function, bowel function domains.
**High Vegetable**			
**Study**	**Results**		**Summary of Direction of Change**
Parsons et al., [26], RCT	Time to progression(TTP): PSA to rise >10 ng/mL, PSAdt or increase in tumour grade or size **Baseline vs. 24 months** HR 0.97 (95% CI 0.76–1.25) (*p* = 0.84).		No statistical difference in time required for rise in PSA > 10 ng/mL, PSAdt or increase in tumour size or grade. Similar numbers of patients from both groups withdrew from the study to pursue active treatment (1.8% vs. 1.9%).
Vegan			
Study	**Results**		**Summary of Direction of Change**
Ornish et al. [27], RCT	**PSA** **Baseline** **I:** 6.32 ± 1.72, **C:** 6.28 ± 1.66 **12 months** **I:** 5.98 ± 1.7, **C:** 6.74 ± 2.1 **Mean change** **I:** −0.25 ± 1.2, **C:** 0.38 ± 1.3		Statistically significant but relatively modest decrease in average of intervention group PSA 0.25 ng/mL (4% of baseline average) and average increase of 0.38 ng/mL (6% of the baseline average) in control group (F = 5.6, *p* = 0.016). PSA change in I vs. C (r = −0.23, *p* = 0.035).
Daubenmier et al., [30], RCT	**QoL (Standard deviation), (*p* value between groups)** **Baseline PCS** **I:** 52.9 (6.8), **C:** 51.7 (8.9) **12 months** **I:** 53.2 (6.6), **C:** 50.2 (9.5), *p* = 0.18 **Baseline MCS** **I:** 51.4 (10.4), **C:** 55.7 (6.6) **12 months** **I:** 50.7 (9.3), **C:** 56.0 (6.7), *p* = 0.01 **Baseline Perceived stress** **I:** 1.16 (0.6), **C:** 0.86 (0.4) **12 months** **I:** 1.16 (0.7), **C:** 0.91 (0.5), *p* = 0.02 **Baseline Sexual function** **I:** 64.9 (20.2), **C:** 63.2 (25.7) **I:** 61.2 (25.6), **C:** 60.2 (28.8), *p* = 0.81		QoL scores were high from baseline to post-study. No significant changes at 12 months as compared to baseline in either arm.
			Intervention arm demonstrated a significant difference to control in perceived stress.
**Low Fat**			
**Study**	**Results**		**Summary of Direction of Change**
Spentzos et al. [38], pre-post study	**PSAdt Median (IQR)** **During step 1:** Estimated 7.6 (5.3–13.8) **During step 2:** 11.3 (6.7, 39.5)		None of the 18 patients had a decline of PSA > 50% from baseline in step 1 or 2. There is a trend towards a delay in PSA progression in step 2 but not statistically significant.
	**Adherence (from food records). Average % of calories from fat intake Baseline:** 36% **First 6 months:** 19% (24% during the first two months, 20% during the next two months)		Food records obtained from 16/18 patients. Only 2/16 patients with average fat consumption constituting <15% of calories. Strict adherence to diet was low.
Thomas et al. [39], pre-post study	**PSA (95% CI)** **Pre-trial:** 24 (13.0–35.1) **Trial:** 30.3 (12.6–48.1) **PSAdt (95% CI)** **Baseline (From 12 months pre-trial mean):** 22.2 (10.5–33.9) **Baseline-withdrawal:** 67.7 (25.6–109.7)		6 out of 7 with early prostate cancer demonstrated stabilisation of PSA. Patients demonstrated a positive difference of 45.5 months in PSAdt across the trial. Statistical significance was not calculated.
	**Quality of life** Scores not reported		No change in their quality of life (QoL), as measured by the Rotterdam Symptom checklist, for the duration of the study.
Low Carbohydrate			
Study	**Results**		**Summary of Direction of Change**
Freedland et al. [28], RCT	**PSA** **Baseline-3 months** **I:** −97 (−98, −95), **C:** −98 (−99, −94), *p* = 0.281 **3–6 months** **I:** −99 (−99.6, −89), **C:** −99 (−99.8, −98), *p* = 0.369		Both arms demonstrated similar degree of % change in PSA. There was no statistically significant different change between arms
	**Adherence (change in homeostatic model assessment (HOMA)Baseline-3 months** **I:** −19 (−54, −1), **C:** 7 (−11, 55), *p* = 0.015 **3–6 months** **I:** −4 (−36, 32), **C:** 36 (−10, 86). *p* = 0.127		Intervention arm decreased by 4% as compared to a 36% increase in the control arm, indicating general adherence to the LCD in intervention arm. However, this was not statistically significant.
Freedland et al. [29], RCT	**PSAdt Months (95% CI)** **Baseline-6 months** **I:** 22 (14–34), **C:** 15 (9–26). *p* = 0.313 **Post hoc analysis (adjusted for hemoconcentration):** **I:** 30 (21–43), **C:** 13 (8–20), *p* = 0.007		Mean PSAdt was similar after intervention between LCD (22 months) and control (15 months, *p* = 0.313) arms. In a post hoc exploratory analysis adjusted for prestudy and baseline PSA, primary treatment, and hemoconcentration, PSAdt prolongation was significantly longer in LCD versus control (30 vs. 13 months, *p* = 0.007) arms.
**Prudent vs. Western**			
**Study**	**Results**		**Summary of Direction of Change**
Yang et al. [31], cohort study	**Overall mortality** **Western [Hazard Ratio (Quartile 4 vs. Quartile1)]:** 2.53 (1.00–6.42) *p* = 0.02 **Prudent [Hazard Ratio (Quartile 4 vs. Quartile1)]:** 0.46 (0.17–1.24) *p* = 0.11 **Cancer-specific mortality** **Western [Hazard Ratio (Quartile 4 vs. Quartile1)]:** 1.67 (1.16–2.42) *p* = 0.01 **Prudent [Hazard Ratio (Quartile 4 vs. Quartile1)]:** 0.64 (0.44–0.93) *p* = 0.02		High post-diagnostic adherence to a Western dietary pattern may increase risks of all-cause mortality and cancer-specific mortality risks.
			High post-diagnostic adherence to a Prudent dietary pattern may lower risks of all-cause mortality and prostate cancer-specific mortality
**High inflammatory, Hyperinsulinaemic, and Insulin-resistant Diets**			
**Study**	**Measure**		**Summary of direction of change**
Langlais et al., [34], Cohort study	**Recurrence** **EDIP ^%^** HR, 1.27; CI, 1.17–1.37 **EDIH** HR, 1.24; CI, 1.05–1.46. **EDIR** HR, 1.22; CI, 1.00–1.48		Positive association between hyperinsulinaemic, high inflammatory, and insulin-resistant diets to time of progression.
			No statistical association between to cancer-specific mortality.

^#^ HA: High adherence; MA: Moderate adherence; LA: Low adherence; ^%^ EDIP: Empirical Dietary Inflammation Pattern; EDIH: Empirical Dietary Index for Hyperinsulinaemia; EDIR: Empirical Dietary Index for Insulin Resistance;: Intervention group; C: Control group; PSA measured in ng/mL; ^ PSAdt measured in months. Direction of change: Green—positive, Red—negative and Yellow—neutral or statistically not significant.

## Data Availability

The authors confirm that the data supporting the findings of this study are available within the article [and/or] its Supplementary Materials. Further enquiries can be directed to the corresponding author.

## Supplementary Materials

The following supporting information can be downloaded at: https://www.mdpi.com/article/10.3390/healthcare13172201/s1, Table S1: Sample search strategy. Table S2: List of full-text article exclusions with reasons.

## Abbreviations

The following abbreviations are used in this manuscript

PCa	Prostate cancer
ADT	Andorgen deprivation therapy
PSA	Prostate Specific Antigen
PSAdt	Prostate Specific Antigen doubling time
HIIT	High intensity interval training
QoL	Quality of Life
