# Supplementary material for: The Impact of Different Dietary Patterns on Mortality and Prognosis After Non-Metastatic Prostate Cancer Diagnosis: A Systematic Review
Source: Healthcare (Basel). 2025 Sep 2;13(17):2201. doi: 10.3390/healthcare13172201 (PMC12428250; doi:10.3390/healthcare13172201)
Supplement: Supplementary file 1 [file healthcare-13-02201-s001.zip › healthcare-3719328-supplementary.pdf]

Supplementary Materials

Supplementary Tables

Supplementary Table S1: Sample search strategy

Embase <1974 to 2024 April 16>

#	Query	Number of records
1	Prostatic Neoplasms/	14571
2	Diet, High-Protein/ or Diet, Ketogenic/ or Diet, Carbohydrate-Restricted/ or FODMAP Diet/ or Diet, High-Protein Low-Carbohydrate/ or Diet, Carbohydrate Loading/ or Diet, Atherogenic/ or Diet/ or Diet, Western/ or Diet, Paleolithic/ or Diet, Macrobiotic/ or Immunonutrition Diet/ or Diet, High-Fat/ or Diet, Fat-Restricted/ or Diet, Plant-Based/ or Diet, Reducing/ or Diet, Vegetarian/ or Diet, Vegan/ or Diet, Diabetic/ or Diet, Mediterranean/ or Diet, Gluten-Free/ or Diet, Sodium-Restricted/ or Diet, Protein-Restricted/ or Diet, Cariogenic/ or Diet, Healthy/	405390
3	Intermittent Fasting/	1475
4	Recurrence/ or Neoplasm Recurrence, Local/ or Recurrence.mp.	914962
5	Mortality/ or Mortality.mp.	1992191

6	Remission, Spontaneous/ or Remission Induction/ or Remission.mp.	378063
7	Progression-Free Survival/ or Disease Progression/ or Progression.mp.	1287064
8	Quality of life.mp. or "Quality of Life"/	843118
9	PSA.mp. or Prostate-Specific Antigen/	122582
10	Prognosis/	692181
11	2 or 3	406328
12	4 or 5 or 6 or 7 or 8 or 9 or 10	5284908
13	prostate cancer/	231562
14	1 or 13	245426
15	11 and 12 and 14	1231
16	limit 15 to english	1212

Supplementary Table S2: List of full-text article exclusions with reasons

Article	Reason for exclusion	Category
Blanchard CM, Courneya KS, Stein K. Cancer survivors' adherence to lifestyle behavior recommendations and associations with health-related quality of life: results from the American Cancer Society's SCS-II. Journal of Clinical Oncology. 2008 May 1;26(13):2198-204.	No relevant primary or secondary outcomes measured. Lifestyle behaviours investigated instead.	No outcomes of interest
Brinkman M, Trumpour S, Lyford K, Gray D. Choosing healthy eating choices and keeping active for men having the androgen therapy experience the Checkmate pilot study. InBJU INTERNATIONAL 2018 Aug 1 (Vol. 122, pp. 16-16). 111 RIVER ST, HOBOKEN 07030-5774, NJ USA: WILEY.	Only conference abstract available	Only conference abstract or protocol available
Frattaroli J, Weidner G, Dnistrian AM, Kemp C, Daubenmier JJ, Marlin RO, Crutchfield L, Yglecias L, Carroll PR, Ornish D.	Results of a previous trial with a focus on clinical events involving treatment, cardiac events, other	No outcomes of interest

Clinical events in prostate cancer lifestyle trial: results from two years of follow-up. Urology. 2008 Dec 1;72(6):1319-23.	cancers and so on. Original trial with relevant outcomes by Ornish included.	
Kenfield SA. Combined impact of diet and lifestyle after diagnosis on risk of prostate cancer death.	Only conference abstract available	Only conference abstract or protocol available
Snyder DC, Sloane R, Lobach D, Lipkus IM, Peterson B, Kraus W, Demark-Wahnefried W. Differences in baseline characteristics and outcomes at 1-and 2-year follow-up of cancer survivors accrued via self-referral versus cancer registry in the FRESH START diet and exercise trial. Cancer Epidemiology Biomarkers & Prevention. 2008 May;17(5):1288-94.	Study design comparing the characteristics and performance of self-referred versus registry-ascertained cancer survivors in a lifestyle intervention trial	Study design outside of inclusion criteria
Parsons JK, Marshall JR, Nelson H. Does altering diet affect progression of prostate cancer? The MEAL study. Bulletin of the American College of Surgeons. 2013 Oct;98(10):57-9.	Study protocol only. Relevant published paper from 2018 included.	Only conference abstract or protocol available
Forslund M, Ottenblad A, Ginman C, Johansson S, Nygren P, Johansson B. Effects of a nutrition intervention on acute and late	No relevant primary or secondary outcomes measured. Outcome measured related to degree of mitigation of	No outcomes of interest

bowel symptoms and health-related quality of life up to 24 months post radiotherapy in patients with prostate cancer: a multicentre randomised controlled trial. Supportive Care in Cancer. 2020 Jul;28(7):3331-42.	nutrition intervention on gastrointestinal-associated side effects and QoL relating specifically to androgen-therapy.	
Jaime* AT, Huynh LM, Derderian R, Choi E, Su HW. PD43-09 Heart-Healthy Diet And High-Intensity Interval Training As A Lifestyle Intervention For Patients With Prostate Cancer Recurrence Following Radical Prostatectomy. The Journal of Urology. 2020 Apr;203(Supplement 4):e903-4.	Only conference abstract available	Only conference abstract or protocol available
Fay-Watt V, O'Connor S, Roshan D, Romeo AC, Longo VD, Sullivan FJ. The impact of a fasting mimicking diet on the metabolic health of a prospective cohort of patients with prostate cancer: a pilot implementation study. Prostate cancer and prostatic diseases. 2022 Mar 21:1-6.	No relevant primary or secondary outcomes measured. Metabolic risk factors investigated instead.	No outcomes of interest

Klement RJ, Sweeney RA. Impact of a ketogenic diet intervention during radiotherapy on body composition: I. Initial clinical experience with six prospectively studied patients. BMC research notes. 2016 Dec;9(1):1-3.	Six patients of different cancers studied. The one prostate cancer patient included was of metastatic status.	Population outside of inclusion criteria
Baguley BJ, Skinner TL, Leveritt MD, Wright OR. The impact of a mediterranean-style dietary pattern on cancer-related fatigue and quality of life in men with prostate cancer treated with androgen deprivation therapy: A pilot study. Asia-Pacific Journal of Clinical Oncology. 2016 Jan 1;12(s7):115-.	Only conference abstract available. Published paper included.	Only conference abstract or protocol available
Lin PH, Howard L, Freedland SJ. Impact of Low Carbohydrate Diet on Self-Report Fatigue and Weakness in Prostate Cancer Patients. The Journal of Urology. 2021 Sep;206(3):499-501.	A narrative of only the results of a trial. No relevant primary or secondary outcomes measured. Self-reported fatigue and weakness investigated instead.	Study design outside of inclusion criteria
Focht BC, Lucas AR, Grainger E, Simpson C, Thomas-Ahner JM, Clinton SK. The Individualized Diet and Exercise Adherence Pilot Trial (IDEA-P) in prostate cancer patients undergoing androgen	Study protocol only. Relevant published paper from 2018 also makes no mention of metastatic status.	Only conference abstract or protocol available

deprivation therapy: study protocol for a randomized controlled trial. Trials. 2014 Dec;15(1):1-9.		
Baguley BJ, Skinner TL, Jenkins DG, Wright OR. Mediterranean-style dietary pattern improves cancer-related fatigue and quality of life in men with prostate cancer treated with androgen deprivation therapy: A pilot randomised control trial. Clinical Nutrition. 2021 Jan 1;40(1):245-54.	Updated 2022 paper investigating the same cohort and with more outcome measures included instead.	Study design outside of inclusion criteria
Hansen RD, Larsen R, Borre M, Friis S, Åman P, Steingrimsdottir L, Hallmans G, Overgaard K, Tjonneland A. Nordic lifestyle intervention trial on prostate cancer progression (NILS). Ineuropean journal of cancer 2012 (Vol. 48, No. S5, pp. S286-S286).	Only conference abstract available	Only conference abstract or protocol available
Cipolla BG, Havouis R, Moulinoux JP. Polyamine reduced diet (PRD) nutrition therapy in hormone refractory prostate cancer patients. Biomedicine & Pharmacotherapy. 2010 May 1;64(5):363-8.	Only includes metastatic prostate cancer patients.	Population outside of inclusion criteria

O'Neill RF, Haseen F, Murray LJ, O'Sullivan JM, Cantwell MM. A randomised controlled trial to evaluate the efficacy of a 6-month dietary and physical activity intervention for patients receiving androgen deprivation therapy for prostate cancer. Journal of Cancer Survivorship. 2015 Sep;9(3):431-40.	Includes unknown staging and metastatic prostate cancer patients (TXa and T4)	Population outside of inclusion criteria
Snyder DC, Morey MC, Sloane R, Stull V, Cohen HJ, Peterson B, Pieper C, Hartman TJ, Miller PE, Mitchell DC, Demark-Wahnefried W. Reach out to ENhancE Wellness in Older Cancer Survivors (RENEW): design, methods and recruitment challenges of a home-based exercise and diet intervention to improve physical function among long-term survivors of breast, prostate, and colorectal cancer. Psycho-Oncology: Journal of the Psychological, Social and Behavioral Dimensions of Cancer. 2009 Apr;18(4):429-39.	Pooled data of 3 different cancers. The included prostate cancer patient is of metastatic status.	Population outside of inclusion criteria

Chi JT, Lin PH, Tolstikov V, Howard L, Chen EY, Bussberg V, Greenwood B, Narain NR, Kiebish MA, Freedland SJ. Serum metabolomic analysis of men on a low-carbohydrate diet for biochemically recurrent prostate cancer reveals the potential role of ketogenesis to slow tumor growth: a secondary analysis of the CAPS2 diet trial. Prostate Cancer and Prostatic Diseases. 2022 Mar 25:1-8.	No relevant primary or secondary outcomes measured. Serum metabolomic analysis conducted instead. Relevant trial included.	No outcomes of interest
Gregg JR, Zhang X, Chapin BF, Ward JF, Kim J, Davis JW, Daniel CR. Adherence to the Mediterranean diet and grade group progression in localized prostate cancer: an active surveillance cohort. Cancer. 2021 Mar 1;127(5):720-8.	No relevant primary or secondary outcomes measured. Outcomes investigated involve progression-free survival investigated.	No outcomes of interest
Chan JM, Holick CN, Leitzmann MF, Rimm EB, Willett WC, Stampfer MJ, Giovannucci EL. Diet after diagnosis and the risk of prostate cancer progression, recurrence, and death (United States). Cancer Causes & Control. 2006 Mar;17(2):199-208.	Observational study investigating effect of dietary components on prostate cancer disease progression. No dietary pattern investigation outlined.	Study design outside of inclusion criteria

Gregg JR, Zheng J, Lopez DS, Reichard C, Browman G, Chapin B, Kim J, Davis J, Daniel CR. Diet quality and Gleason grade progression among localised prostate cancer patients on active surveillance. British journal of cancer. 2019 Feb;120(4):466-71.	Food frequency questionnaire used to investigate diet quality of Gleason Grade progression. No dietary pattern or relevant primary or secondary outcomes investiated.	Study design outside of inclusion criteria
Carmody J, Olendzki B, Reed G, Andersen V, Rosenzweig P. A dietary intervention for recurrent prostate cancer after definitive primary treatment: results of a randomized pilot trial. Urology. 2008 Dec 1;72(6):1324-8.	No mention of metastatic status.	Population outside of inclusion criteria
Erdrich S, Bishop KS, Karunasinghe N, Han DY, Ferguson LR. A pilot study to investigate if New Zealand men with prostate cancer benefit from a Mediterranean-style diet. PeerJ. 2015 Jul 2;3:e1080.	DNA damage, C-reactive protein (CRP) and PSA were measured. Isolated PSA measurements and comparisons were inadequately reported.	No outcomes of interest
Mohamad H, Ntessalen M, Craig LC, Clark J, Fielding S, N'Dow J, Heys SD, McNeill G. A self-help diet and physical activity intervention with dietetic support for weight management in men treated for prostate cancer: pilot study of the Prostate Cancer	Self-help diet and activity-based intervention with telephone-delivered advice and resources from a dietitian. No clear dietary pattern outlines in intervention.	Study design outside of inclusion criteria

Weight Management (PRO-MAN) randomised controlled trial. British Journal of Nutrition. 2019 Sep;122(5):592-600.		
Kumar NB, Cantor A, Allen K, Riccardi D, Besterman-Dahan K, Seigne J, Helal M, Salup R, Pow-Sang J. The specific role of isoflavones in reducing prostate cancer risk. The Prostate. 2004 May 1;59(2):141-7.	Study design does not fit the criteria: Dietary pattern not investigated, only isoflavone supplementation.	Study design outside of inclusion criteria
Parsons J, Zarieh D, Pierce J, Mohler J, Paskett E, Hansel D, Kibel A, Hahn O, Taylor J, Grubb R, Stroup S. LBA19 The Men's Eating and Living (MEAL) Study (CALGB 70807 [Alliance]): a randomized clinical trial of a diet intervention in men on active surveillance for prostate cancer. The Journal of Urology. 2018 Apr;199(4S):e1077-.	Feasability trial. No relevant primary or secondary outcomes measured.	No outcomes of interest
Pettersson A, Nygren P, Persson C, Berglund A, Turesson I, Johansson B. Effects of a dietary intervention on gastrointestinal symptoms after prostate cancer radiotherapy: long-term results	No relevant primary or secondary outcomes measured. Outcome measured related to degree of mitigation of nutrition intervention on gastrointestinal-associated side effects and QoL relating specifically to androgen-	No outcomes of interest. Population outside

from a randomized controlled trial. Radiotherapy and Oncology. 2014 Nov 1;113(2):240-7.	therapy. Staging of 9 prostate cancer patients unknown.	of inclusion criteria
Dalais FS, Meliala A, Wattanapenpaiboon N, Frydenberg M, Suter DA, Thomson WK, Wahlqvist ML. Effects of a diet rich in phytoestrogens on prostate-specific antigen and sex hormones in men diagnosed with prostate cancer. Urology. 2004 Sep 1;64(3):510-5.	Study design does not fit the criteria: Dietary pattern not investigated, only soy supplementation.	No outcomes of interest
Aronson WJ, Barnard RJ, Freedland SJ, Henning S, Elashoff D, Jardack PM, Cohen P, Heber D, Kobayashi N. Growth inhibitory effect of low fat diet on prostate cancer cells: results of a prospective, randomized dietary intervention trial in men with prostate cancer. The Journal of urology. 2010 Jan;183(1):345-50.	No relevant primary or secondary outcomes measured. No mention of metastatic status.	No outcomes of interest. Population outside of inclusion criteria
Demark-Wahnefried W, Robertson CN, Walther PJ, Polascik TJ, Paulson DF, Vollmer RT. Pilot study to explore effects of low-fat, flaxseed-supplemented diet on proliferation of benign prostatic	Pooled data of 2 different cancers. The some of the included prostate cancer patients were of unknown metastatic status.	Population outside of inclusion criteria

epithelium and prostate-specific antigen. Urology. 2004 May 1;63(5):900-4.		
Hébert JR, Hurley TG, Harmon BE, Heiney S, Hebert CJ, Steck SE. A diet, physical activity, and stress reduction intervention in men with rising prostate-specific antigen after treatment for prostate cancer. Cancer epidemiology. 2012 Apr 1;36(2):e128-36.	Dietary intervention involved parallel nutrition advice to exercise intervention. No dietary pattern outlined. No mention of metastatic status.	Study design outside of inclusion criteria.
Di Maso M, Augustin LS, Toffolutti F, Stocco C, Dal Maso L, Jenkins DJ, Fleshner NE, Serraino D, Polesel J. Adherence to mediterranean diet, physical activity and survival after prostate cancer diagnosis. Nutrients. 2021 Jan 16;13(1):243.	No mention of metastatic status.	Population outside of inclusion criteria
Blackie K, Bobe G, Takata Y. Vegetarian diets and risk of all-cause mortality in a population-based prospective study in the United States. Journal of Health, Population and Nutrition. 2023 Nove 23;42(1):130.	Pooled data of patients from Prostate, Lung, Colorectal, Ovarian Cancer Screening Trial cohort study.	Population outside of inclusion criteria

Bourke L, Gilbert S, Hooper R, Steed LA, Joshi M, Catto JW, Saxton JM, Rosario DJ. Lifestyle changes for improving disease-specific quality of life in sedentary men on long-term androgen-deprivation therapy for advanced prostate cancer: a randomised controlled trial. European urology. 2014 May 1;65(5):865-72.	Dietary intervention involved parallel nutrition advice to exercise intervention. No dietary pattern outlined or significant changes to diet recorded from baseline.	Study design outside of inclusion criteria
Chi JT, Lin PH, Tolstikov V, Howard L, Chen EY, Bussberg V, Greenwood B, Narain NR, Kiebish MA, Freedland SJ. Serum metabolomic analysis of men on a low-carbohydrate diet for biochemically recurrent prostate cancer reveals the potential role of ketogenesis to slow tumor growth: a secondary analysis of the CAPS2 diet trial. Prostate Cancer and Prostatic Diseases. 2022 Dec;25(4):770-7.	Secondary analysis of PSADT. Relevant primary analysis of the RCT is already included.	No outcomes of interest
Van Blarigan EL, Chan JM, Sanchez A, Zhang L, Winters-Stone K, Liu V, Macaire G, Panchal N, Graff RE, Tenggara I, Luke A. Protocol for a 4-arm randomized controlled trial testing remotely delivered exercise-only, diet-only, and exercise+ diet interventions	Protocol only.	Only conference abstract or protocol available

among men with prostate cancer treated with radical prostatectomy (Prostate 8-II). Contemporary Clinical Trials. 2023 Feb 1;125:107079.		
Tulipan J, Kofler B. Implementation of a Low-Carbohydrate Diet Improves the Quality of Life of Cancer Patients–An Online Survey. Frontiers in Nutrition. 2021 Aug 11;8:661253.	Pooled data of various cancer patients. No specific analysis of non-metastatic prostate cancer patients.	Population outside of inclusion criteria
Thompson AS, Tresserra-Rimbau A, Karavasiloglou N, Jennings A, Cantwell M, Hill C, Perez-Cornago A, Bondonno NP, Murphy N, Rohrmann S, Cassidy A. Association of healthful plant-based diet adherence with risk of mortality and major chronic diseases among adults in the UK. JAMA Network Open. 2023 Mar 1;6(3):e234714-.	Pooled data of various cancer patients. No mention of metastatic status.	Population outside of inclusion criteria
Mosher CE, Sloane R, Morey MC, Snyder DC, Cohen HJ, Miller PE, Demark-Wahnefried W. Associations between lifestyle factors and quality of life among older long-term breast, prostate, and colorectal cancer survivors. Cancer: Interdisciplinary	Pooled data of various cancer patients. No mention of metastatic status. Interview-based assessment of association between lifestyle factors such as diet on quality of life on long-term cancer survivors.	Study design outside of inclusion criteria

International Journal of the American Cancer Society. 2009 Sep 1;115(17):4001-9.		
Liu VN, Van Blarigan EL, Zhang L, Graff RE, Langlais C, Cowan JE, Carroll P, Chan JM, Kenfield SA. Associations between plant-based diets and risk of disease progression in men with prostate cancer.	Only supplementary information. Full article included.	Only conference abstract or protocol available
